# Procoagulant and Antimicrobial Effects of Chitosan in Wound Healing

**DOI:** 10.3390/ijms22137067

**Published:** 2021-06-30

**Authors:** Chih-Hsin Wang, Juin-Hong Cherng, Chuan-Chieh Liu, Tong-Jing Fang, Zhi-Jie Hong, Shu-Jen Chang, Gang-Yi Fan, Sheng-Der Hsu

**Affiliations:** 1 Division of Plastic and Reconstructive Surgery, Department of Surgery, Tri-Service General Hospital, National Defense Medical Center, Taipei 114, Taiwan; super-derrick@yahoo.com.tw; 2 Department and Graduate Institute of Biology and Anatomy, National Defense Medical Center, Taipei 114, Taiwan; i72bbb@gmail.com; 3 Graduate Institute of Life Sciences, National Defense Medical Center, Taipei 114, Taiwan; 4 Department of Cardiology, Cardinal Tien Hospital, New Taipei City 231, Taiwan; chuanchiehliu@gmail.com; 5 Department of Physiology and Biophysics, Graduate Institute of Physiology, National Defense Medical Center, Taipei 114, Taiwan; wonderfulactioncom@gmail.com; 6 Division of Traumatology, Department of Surgery, Tri-Service General Hospital, National Defense Medical Center, Taipei 114, Taiwan; lgf670822@mail.ndmctsgh.edu.tw; 7 Division of Rheumatology/Immunology/Allergy, Department of Internal Medicine, Tri-Service General Hospital, National Defense Medical Center, Taipei 114, Taiwan; belle661011@gmail.com; 8 Laboratory of Adult Stem Cell and Tissue Regeneration, National Defense Medical Center, Taipei 114, Taiwan; u9310318@gmail.com; 9 Division of Urology, Department of Surgery, Tri-Service General Hospital, National Defense Medical Center, Taipei 114, Taiwan

**Keywords:** antimicrobial, chitosan, hemostasis, microbiota, procoagulant, wound dressing

## Abstract

Chitosan, a polysaccharide derived from chitin, has excellent wound healing properties, including intrinsic antimicrobial and hemostatic activities. This study investigated the effectiveness of chitosan dressing and compared it with that of regular gauze dressing in controlling clinically surgical bleeding wounds and profiled the community structure of the microbiota affected by these treatments. The dressings were evaluated based on biocompatibility, blood coagulation factors in rat, as well as antimicrobial and procoagulant activities, and the microbial phylogenetic profile in patients with abdominal surgical wounds. The chitosan dressing exhibited a uniformly fibrous morphology with a large surface area and good biocompatibility. Compared to regular gauze dressing, the chitosan dressing accelerated platelet aggregation, indicated by the lower ratio of prothrombin time and activated partial thromboplastin time, and had outstanding blood absorption ability. Adenosine triphosphate assay results revealed that the chitosan dressing inhibited bacterial growth up to 8 d post-surgery. Moreover, 16S rRNA-based sequencing revealed that the chitosan dressing effectively protected the wound from microbial infection and promoted the growth of probiotic microbes, thereby improving skin immunity and promoting wound healing. Our findings suggest that chitosan dressing is an effective antimicrobial and procoagulant and promotes wound repair by providing a suitable environment for beneficial microbiota.

## 1. Introduction

In recent years, attempts have been made to develop functional biomaterials for the regeneration of tissue damaged or lost due to disease or injury. Choosing and designing suitable biomaterials for tissue engineering are important tasks because they are expected to assist in cellular activities and mimic the natural microenvironment of the tissue, thereby supporting the biological repair process [1,2]. Amongst a wide range of biomaterials, natural polymers, such as chitosan, collagen, alginate, silk, cellulose, hyaluronic acid, and some polynucleotides, are potential candidates for tissue engineering due to their excellent biocompatibility and extracellular matrix (ECM)-mimicking characteristics [3,4].

Chitosan is a linear polysaccharide derived from the partial deacetylation of chitin, which is the second most abundant natural polymer consisting of 2-acetamido-2-deoxy-β-D-glucose that confers a pH-dependent positive charge to the polymer [5]. The high positive charge on chitosan can stimulate erythrocyte adhesion, fibrinogen adsorption, and platelet activation, rendering it an excellent hemostatic agent [6]. Chitosan is permeable to oxygen, promotes immunity, and exhibits characteristics of high biocompatibility and biodegradability, non-antigenicity, low toxicity, and antimicrobial efficacy, indicating its potential in tissue engineering and biomedical applications [7,8,9].

During skin regeneration, the wound healing process comprises complex overlapping phases, which involve components of the blood, extracellular components, and cells [10]. The wound is susceptible to infection and can be an entry point for systemic infections, resulting in the failure of wound repair. Pathogenic organisms, such as staphylococci and streptococci, are the most encountered bacteria causing severe septicity, ranging from skin and soft tissue infections to severe persistent infections [11,12]. Consequently, the treatment of skin lesions requires a dressing that not only stimulates the healing of damaged skin but also provides antimicrobial protection [13]. Chitosan composites have outstanding antimicrobial activity owing to the electrostatic interactions between the protonated NH^3+^ chitosan groups and negatively charged cell membranes of microbes [14,15,16]. Chitosan-based dressings have been shown to possess significant potential as wound dressings in wound repair owing to their high porosity, ability to mimic the ECM of the skin, good superficial contact, and excellent antimicrobial properties [17].

Many studies have been conducted on the application of chitosan and its derivatives in wound treatment due to their intrinsic antimicrobial and wound healing effects [18,19,20,21]. However, understanding the multifaceted host–microbiota interactions and how the wound environment is modulated by the chitosan is essential for developing treatment strategies specifically targeting the corresponding microbiota, and to increase its application in the medicine, food, and cosmetic fields. In this study, we aimed to investigate the effectiveness of chitosan dressing in controlling bleeding wounds, compared with that of regular gauze, as well as to profile the community structure of the microbiota affected by these treatments. To the best of our knowledge, this is the first study to evaluate the antimicrobial effect of pure chitosan dressing on microbiota environment in clinically surgical wounds by performing 16S rRNA-based sequencing.

## 2. Results and Discussion

### 2.1. Characterization of Chitosan Dressing and Biocompatibility Analysis

**Figure 1:**
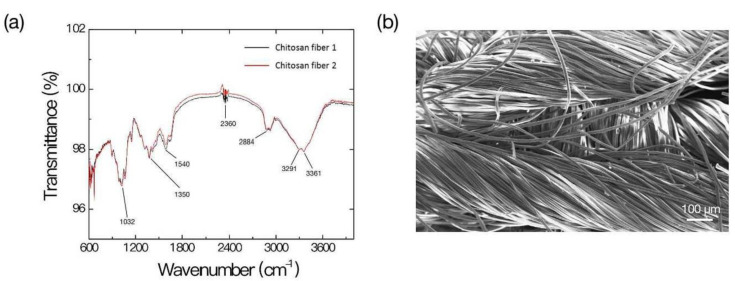
Characterization of the chitosan dressing. (**a**) Fourier-transform infrared spectroscopy results; (**b**) Scanning electron microscopy observation.

The structural characteristics of chitosan dressing used in this study were examined using Fourier-transform infrared (FTIR) spectroscopy. As shown in Figure 1a, the chitosan dressing demonstrated characteristic transmittance bands C–O–C (at 1032 cm^−1^), C–N–H (at 1350 cm^−1^, 1540 cm^−1^), O=C=O vibration (at 2360 cm^−1^), C–H (at 2884 cm^−1^, 3361 cm^−1^), and O–H and N–H bonds (at 3291 cm^−1^, 3361 cm^−1^, respectively), which represented the main features of chitosan [22,23,24,25]. Scanning electron microscopy (SEM) revealed that the chitosan dressing displayed a uniformly fibrous conformation with large surface area (Figure 1b). These features are required for an ideal wound dressing system to accelerate wound hemostasis by absorbing excess drainage, allow oxygen permeation and fluid exchange, and maintaining a suitable environment at the wound–dressing interface [26].

**Figure 2:**
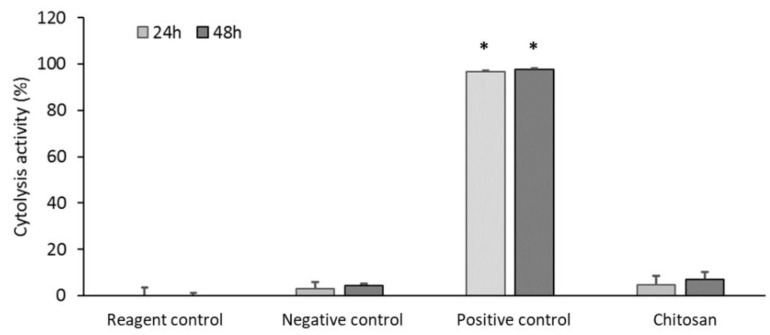
Biocompatibility of chitosan dressing (test product) evaluated through cytolysis activity measurement (* *p* < 0.05; 24 and 48 h incubation).

The absence of adverse reactions in the living system or a suitable host response to the presence of a material is essential for preventing impaired healing. We assessed the biocompatibility of the chitosan dressing by evaluating the cytolysis of WS1 fibroblasts incubated with the dressing for 24 and 48 h. The chitosan dressing could significantly inhibit cytolysis (disintegration of cells) (Figure 2), nearly similar to the reagent or negative control, which are represented by Dulbecco’s modified Eagle’s medium (DMEM) and phosphate-buffered saline (PBS), respectively, thus demonstrating good biocompatibility.

### 2.2. Coagulation Cascade Analysis

**Figure 3:**
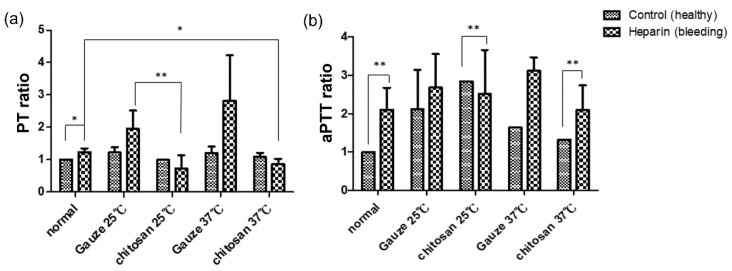
Effects of chitosan and regular gauze dressings on (**a**) prothrombin time (PT) and (**b**) activated partial thromboplastin time (aPTT) (* *p* < 0.05, ** *p* < 0.01).

The disruption of initial blood coagulation in the wound area plays a vital role in impairing the wound healing response [27]. Poor fibrin deposition might result an inability to embed the platelet plug to the surrounding tissues, further distributing wound expansion caused by edema [27]. Hence, the effects of wound dressing on the coagulation cascade must be evaluated. Prothrombin time (PT) and activated partial thromboplastin time (aPTT) represent the time from the activation of coagulation to the generation of fibrin after the initiation of the extrinsic and intrinsic pathways, respectively [28]. A prolonged PT or aPTT may indicate the deficiency of blood coagulation factors. We compared the effect of chitosan and regular gauze dressings on PT and aPTT of rat blood at 25 °C and 37 °C, which represent the room temperature and human or rat body temperature, respectively. As shown in Figure 3, the chitosan dressing displayed the lowest PT and aPTT ratio both in healthy rats and rats with heparin consumption, especially at 37 °C. Thus, the effectiveness of chitosan in accelerating coagulation was established.

**Figure 4:**
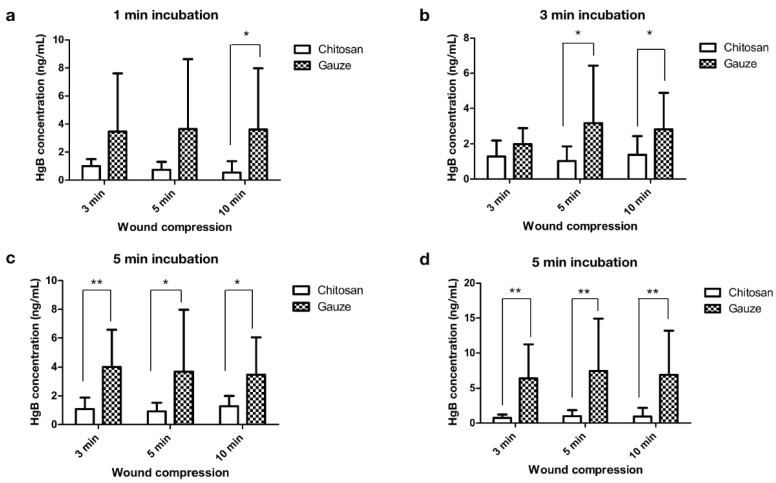
Hemoglobin concentration of chitosan and regular gauze dressings in contact with abdominal surgical wounds (3, 5, and 10 min wound compression) in normal saline solution incubated for (**a**) 1, (**b**) 3, (**c**) 5, and (**d**) additional 5 min (* *p* < 0.05, ** *p* < 0.01).

To evaluate the effect of the dressings on blood coagulation, both dressings were used to compress abdominal surgical wounds of patients for 3, 5, and 10 min, and the ability of the dressing to absorb blood was analyzed by performing the hemoglobin assay. The wound-contact dressings were incubated in saline solution for 1, 3, 5, and 10 min. The residual hemoglobin concentration of the wound-contact chitosan dressing in the saline solution was significantly lower than that in the regular gauze dressing at all incubation times (Figure 4), indicating that chitosan acted as an effective procoagulant activator that helps the blood to clot faster [19]. Studies have reported that chitosan alters the microstructure of hemoglobin and increases the viscosity of blood by interacting with negatively charged thrombocytes and erythrocytes [29,30]. In addition, chitosan molecules are more effective than chitin during blood coagulation, by not merely accelerating platelet aggregation but also erythrocyte aggregation [31].

### 2.3. Antimicrobial Activities

**Figure 5:**
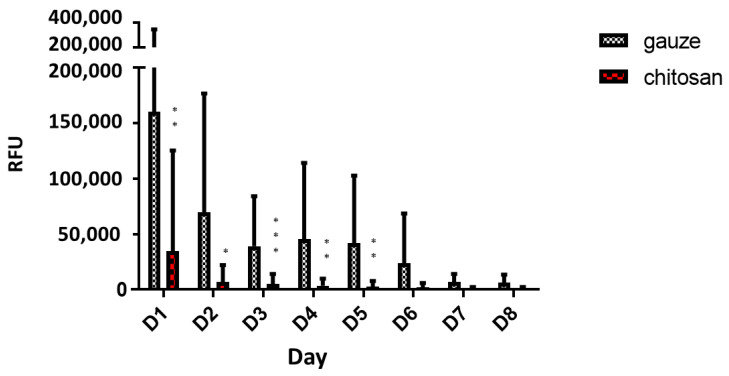
Adenosine triphosphate (ATP) assay for determining bacterial growth in patient wound in contact with chitosan and regular gauze dressings for 8 d post-surgery (RFU = relative fluorescence units, * *p* < 0.05, ** *p* < 0.01, *** *p* < 0.001).

The wound environment is conducive to microbial growth as it contains microbes, micronutrients, and exudates [32]. Infection that may result from inadequate wound care triggers the systemic immune response and impedes vital processes involved in wound healing. Hence, the ability of the wound dressing to protect against microbes is important. We compared the antimicrobial activity of dressings in contact with wounds up to 8 d post-surgery. Compared with the gauze dressing, the chitosan dressing was found to significantly inhibit bacterial growth in the wound during the first 5 d post-surgery (Figure 5), indicating the strong antimicrobial characteristic of chitosan. This trend continued as time progressed, but no statistical difference was observed between treatments after 5 d post-surgery. Thus, chitosan dressing is a more efficient antimicrobial than regular gauze, especially shortly after wounding. Effective microbial protection at the onset of wound healing is critical in determining whether the wound is likely to heal, thereby reducing patient trauma, treatment costs, and the demand for resources in wound management [33]. The excellent antimicrobial activity of chitosan is attributed to the presence of numerous basic amino groups, with an overall cationic charge at an acidic pH, which helps in the disruption and lysis of bacterial cells [20,34,35].

**Figure 6:**
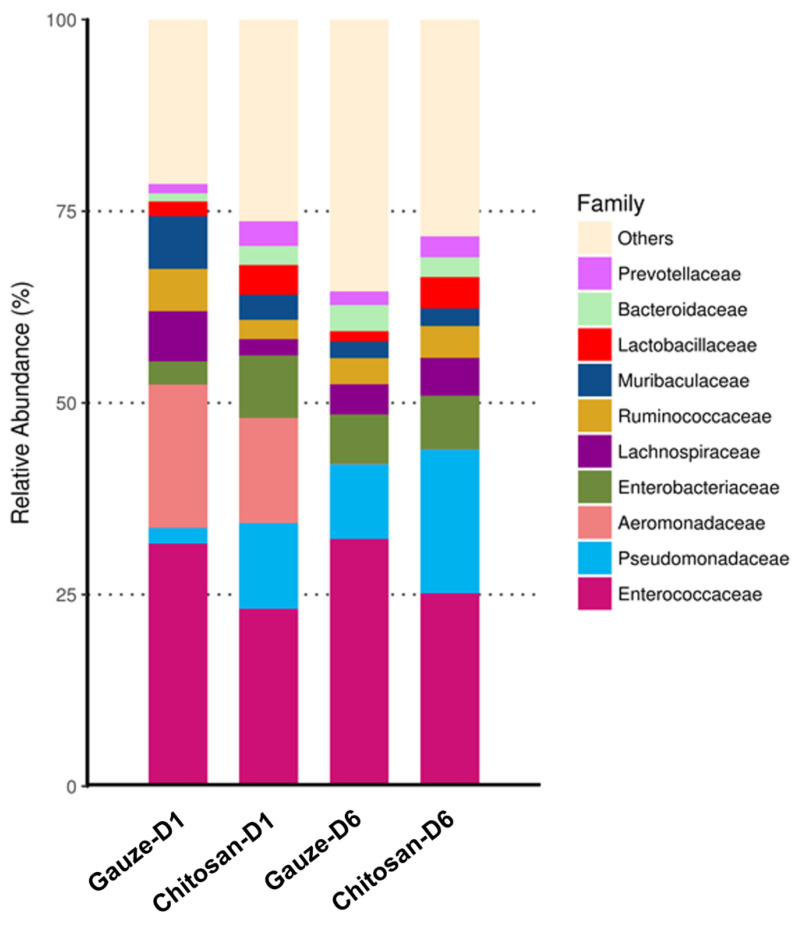
Relative abundance of most predominant bacteria identified in wound-contact chitosan and regular gauze dressings up to 6 d post-surgery (D1 = day 1, D6 = day 6).

**Figure 7:**
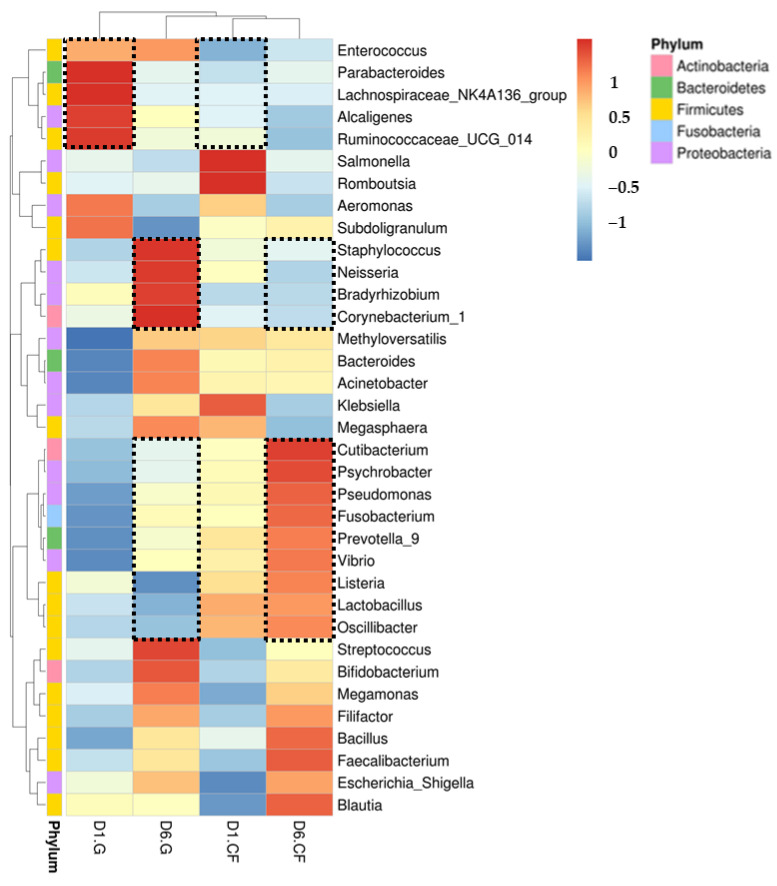
Heat-map and hierarchical clustering of genera of bacterial distribution in wound-contact chitosan and regular gauze dressings up to 6 d post-surgery. Black dash indicates significant difference between the groups (D1 = day 1, D6 = day 6, G = gauze, CF = chitosan).

To evaluate the phylogenetic profile of the microbial community in the wound affected by the dressing treatments, 16S rRNA-based sequencing on the Illumina MiSeq platform was performed. Figure 6 demonstrates the influence of wound dressing on the relative abundance of bacterial population at the family level. We found that the chitosan dressing inhibited the growth of members of *Enterobacteriaceae* (from 8.1% in 1 d to 7% in 6 d post-surgery) and *Muribaculaceae* (from 3.3% in 1 d to 2.3% in 6 d post-surgery) and promoted the growth of members of *Pseudomonadaceae* by 7.6% up to 6 d post-surgery compared with the regular gauze dressing. In both dressings, the abundance of *Enterococcaceae* was found to be the highest among all families and increased at 6 d post-surgery; however, it was less in the chitosan dressing than that in regular gauze at days 1 (31.6% vs. 23.1%) and 6 (32.3% vs. 25.2%) post-surgery. In addition, *Aeromonadaceae* showed a lower abundance in the wound with the chitosan-dressing treatment (13.7%) at 1 d post-surgery compared to the regular gauze dressing (18.7%), and after 6 d post-surgery, *Aeromonadaceae* was not observed in both treatments. Furthermore, a heat-map analysis was conducted according to species annotation and abundance information of all groups in genus hierarchy (Figure 7). *Enterococcus*, *Parabacteroides*, *Lachnospiraceae*, *Alcaligenes*, and *Ruminococcaceae* were abundant in wounds treated with regular gauze dressing at 1 d post-surgery and were replaced with *Staphylococcus*, *Neisseria*, *Bradyrhizobium*, and *Corynebacterium* after 6 d; however, these bacteria were not identified in wounds treated with the chitosan dressing. In addition, wounds treated with the chitosan dressing showed an abundance of *Cutibacterium*, *Psychrobacter*, *Pseudomonas*, *Fusobacterium*, *Prevotella*, *Vibrio*, *Listeria*, *Lactobacillus*, and *Oscillibacter* at 6 d post-surgery.

An infection occurs when virulence factors expressed by microorganisms in a wound overtake the natural immune system of the host, thus initiating their invasion and dissemination, resulting in a series of local and systemic responses [33]. Postoperative wound infections and skin and soft-tissue infections are most often caused by biofilm-forming bacteria, including staphylococci, *Enterococcus* spp., *Enterobacter* spp., *Peptostreptococcus* spp., *Acinetobacter baumannii*, *Escherichia coli*, and *Parabacteroides* [33,36,37,38]. Our findings suggest that treating surgical wounds with chitosan dressings can prevent the growth of pathogenic species that may initiate wound infection. In addition, some common probiotics, such as *Prevotella*, *Lactobacillus*, and *Oscillibacter*, were present in wounds with chitosan dressing up to 6 d post-surgery but were absent in wounds with regular gauze dressing. Probiotics are microbes beneficial to the host and can positively promote wound healing by acting as signaling receptors against pathogens and stimulating the production of beta-defensins, which improve skin immunity [39,40].

**Figure 8:**
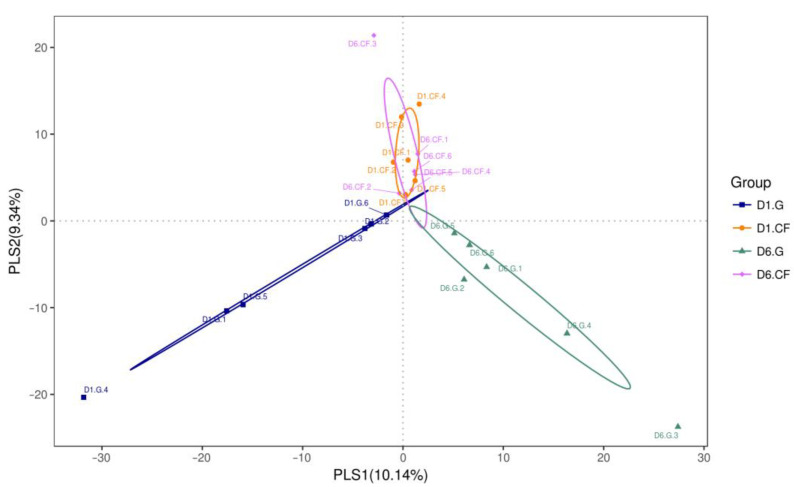
Partial least squares discriminant analysis (PLS-DA) of bacteria identified in wound-contact chitosan and regular gauze dressings for up to 6 d post-surgery. PLS1 and PLS2 represent 10.14% and 9.34% of variance, respectively. (D1 = day 1; D6 = day 6; G = gauze; CF = chitosan).

Partial least squares discriminant analysis (PLS-DA) was performed by analyzing the operational taxonomic unit (OTU) of bacteria from all dressing samples to evaluate compositional differences and similarities between bacterial populations in the wound after the application of the dressing treatment. The PLS-DA scatterplot displayed structural variability of 19.48% (PLS1: 10.14%; PLS2: 9.34%) in the bacterial communities between the different treatments (Figure 8). The microbial composition in the wound was altered following regular gauze-dressing treatment at 1 d and 6 d post-surgery, shown as blue and green ellipses in Figure 8, respectively, which clustered separately. Conversely, bacterial composition following the chitosan-dressing treatment was almost similar at 1 d and 6 d post-surgery, depicted with orange and pink ellipses in Figure 8, respectively, with overlapping clusters. In addition, microbial populations varied between the two dressing treatments at both 1 and 6 d post-surgery. The PLS-DA results confirm the results from heat-map analysis that alterations in the bacterial community with regular gauze-dressing treatment were more significant than those with chitosan-dressing treatment [41,42].

**Figure 9:**
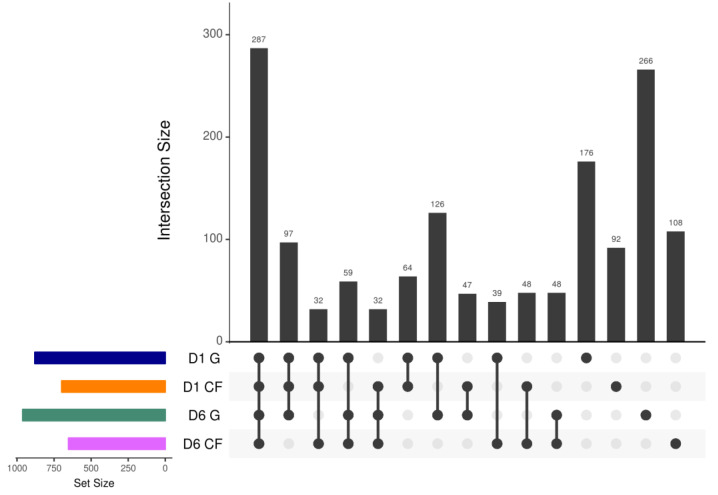
UpSet plot of intersections between sets of bacterial genes induced (family-wise error rate <0.05, log 2-fold change >2) by chitosan-dressing treatment compared with gauze-dressing treatment. The bar chart on the left indicates the total number of upregulated bacterial genes for each dressing. The upper bar chart indicates the intersection size between sets of bacterial genes upregulated with one or more dressings. Dark connected dots in the bottom panel represent the substrates considered for each intersection (D1 = day 1; D6 = day 6; G = gauze; CF = chitosan).

The variability and similarity of bacterial populations in both dressings were also observed by analyzing the intersection size among sets of bacterial genes using the UpSet plot analysis (Figure 9). Genes in bacterial communities clustered into 1521 orthogroups [43]. The results revealed that 287 orthogroups were defined as the largest orthogroups, indicating that orthogroups in wounds with chitosan dressing after 6 d of treatment were shared and upregulated to the orthogroups in the other three treatment groups. Moreover, 266 and 176 orthogroups were specifically upregulated in the wound after 6 d and 1 d of treatment using regular gauze dressing, respectively. These results were quite consistent with the results of PLS-DA.

Thus, chitosan dressing can effectively protect the wound from microbial infection as well as improve the composition of the microbial community to facilitate wound repair.

## 3. Materials and Methods

### 3.1. Experimental Dressing

Surgical cotton gauze was purchased from China Surgical Dressings Company (Taipei, Taiwan (R.O.C.)).

The chitosan dressing was produced by using a wet-spinning method [19]. Chitosan raw material (Mw = 100 kDa, degree of deacetylation ≥ 85%) was purchased from Une Shin Trading Co., Ltd. (CAS No. 9012–76–4; New Taipei City, Taiwan). This material was dissolved in 3% (*v*/*v*) and 5% (*w*/*v*) concentration of acetic acid by stirring overnight at 25 °C. The solution was then diluted with methanol to reach 3% (*w*/*v*) final solution concentration, followed by solution filtering through a cloth filter in an ultrasonic bath to remove the air bubbles. Further, the solution was injected into a coagulation bath maintained at 40 °C containing a 10% solution of 1 M NaOH in distilled water. The fibers were allowed to form in this medium for 1 d, followed by washing with distilled water several times. Further, the fibers were suspended in aq. 2% TWEEN20 for 5 min, followed by 50%, 60%, and 70% methanol soaking for 5 min, respectively. The obtained chitosan filaments were thus compressed to drain the absorbed liquid on the mangled machine and were dried in the oven at 60 °C in a mold. Finally, the chitosan dressing was sterilized using gamma radiation of 25 kGy before use.

### 3.2. Characterization of Chitosan Dressing

The structural characteristics of the chitosan dressing were examined using an FTIR spectrometer (Nicolet 8700, Thermo Scientific, Waltham, MA, USA) at 600–4000 cm^−1^. For each measurement, 32 scans/spectrum were coded at 1 cm^−1^ resolution. Furthermore, the surface morphology of the chitosan dressing was observed using SEM (Hitachi S–3000N, Hitachi High Technologies, Krefeld, Germany), and the SEM images were obtained at 500× to 1000× magnification under an accelerating voltage of 1.5 kV at a working distance of ~15.0 mm.

### 3.3. Biocompatibility Analysis

To evaluate the biocompatibility of the chitosan dressing, human skin fibroblast cells (WS1, ATCC number: CRL–1502), at a density of 10^5^ cells/mL, were loaded in direct contact with the dressing and incubated in a humidified incubator at 37 °C with 5% CO_2_. In addition to evaluating the dressing (test product), the reagent control, negative control, and positive control groups, which represent DMEM, PBS, and 10% dimethyl sulfoxide (DMSO), respectively, were evaluated for comparison. The samples were cultured in DMEM supplemented with 10% (*v*/*v*) fetal bovine serum and 1 U/mL streptomycin–penicillin for 24 and 48 h. Cytolysis activity was investigated using the colorimetric 3-(4,5-dimethylthiazol-2-yl)-2,5-diphenyltetrazolium bromide (MTT) assay. The medium from each group was removed and replaced with 20 μL MTT (5 mg/mL) and incubated for 4 h at 37 °C, followed by the addition of DMSO for 10 min. The absorbance was read at 570 nm on a microplate reader (Bio-Tek ELX-800; BioTek, Winooski, VT, USA). The tests were conducted in triplicate.

### 3.4. PT and aPTT Analysis

The animal study was approved by the Institutional Animal Care and Use Committee (IACUC-19-174) at the National Defense Medical Center (Taipei, Taiwan). Twelve 8-week-old male Sprague–Dawley rats were purchased from Bio-LASCO Co. Ltd. (Taipei, Taiwan) and divided into two groups, namely healthy rats (*n* = 6) and rats with heparin consumption (*n* = 6). Blood samples were collected from both groups and transferred to vials containing 3.2% (*w*/*v*) sodium citrate. The blood was then incubated with the chitosan or regular gauze dressing at 25 °C and 37 °C; blood without dressing material served as the control and was subjected as ratio. For PT and aPTT analysis, the dressings were removed, and sera were collected and fed into an automated blood hemostasis analyzer (CS-2100i; Sysmex Corp., Kobe, Japan). Each experiment was performed in triplicate.

### 3.5. Hemoglobin Absorption Analysis

The clinical trial conducted in this study was approved by the Institutional Review Board (IRB No. 1-108-05-083) of the Tri-Service General Hospital (Taipei, Taiwan) and was registered at the US National Institute of Health Clinical Trials Registry (https://clinicaltrials.gov/ct2/show/NCT04884919 (accessed on 1 May 2021)). Each dressing was used to swab the wound incisions of patients undergoing abdominal surgery (*n* = 30) for 3, 5, and 10 min of manual compression. Each patient received two dressing treatments at the same time, and both dressings were applied to the same wound. One half of the wound was covered with the chitosan dressing and the other half with regular gauze. The dressings were then placed in jars containing 0.9% (*w*/*v*) normal saline for 1, 3, 5, and 10 min. The optical density of 1 mL of each solution was measured at 540 nm wavelength. Hemoglobin concentration was evaluated by interpolating from a standard curve of hemoglobin (H7379, Sigma-Aldrich; Merck KGaA, Darmstadt, Germany). The tests were conducted in triplicate.

### 3.6. Antimicrobial Test

Each dressing was used to swab wound incisions of patients undergoing abdominal surgery up to 8 d post-surgery. The number of bacteria in the dressings was evaluated using the ATP bioluminescence assay, expressed in relative fluorescence units (RFUs), using an ATP luminometer (LuciPac Pen PD 30, Kikkoman Biochemifa Co., Tokyo, Japan).

### 3.7. Phylogenetic Analysis by 16S-rRNA PCR Amplification and Sequencing

Each dressing in contact with wound incisions of patients undergoing abdominal surgery at days 1 and 6 post-surgery was collected and kept at 4 °C within an hour for genomic DNA isolation. Total DNA was isolated from the samples using the DNeasy Blood & Tissue kit (QIAGEN, Germany). Distinct regions (V3-V4) of 16S rRNA genes were amplified using the specific primers 16S V3+V4: 314F-806R with the barcode. All PCR reactions were carried out with the Phusion^®^ High-Fidelity PCR Master Mix (New England Biolabs). Amplicon sequencing was then performed by using 300 bp paired-end raw reads, and all paired-end reads were assembled using FLASH v.1.2.7 [44]. As a quality control, low-quality reads (Q < 20) were discarded in the QIIME 1.9.1 pipeline [45]. To further increase the group distinction, the supervised partial-least-squares discriminant analysis (PLS-DA) was used to evaluate and visualize variance based on OTUs level of gut microbiota composition among the groups. PLS-DA was performed using the R package mixOmics.

### 3.8. Statistical Analysis

The data are presented as mean ± standard error of the mean. Data mean were compared by one-way ANOVA. The significance level was set at * *p* < 0.05, ** *p* < 0.01, and *** *p* < 0.001. Statistical calculations were performed using SPSS software version 21 (SPSS, Chicago, IL, USA). Significance of all species among groups at various taxonomic level were detected using differential abundance analysis with a zero-inflated Gussian (ZIG) log-normal model as implemented in the “fitFeatureModel” function of the Bioconductor metagenomeSeq package [46]. ANOSIM and MRPP analysis were used to determine whether the community structures significantly differ among and within groups.

## 4. Conclusions

In summary, our findings reveal the superior procoagulant and antimicrobial properties of chitosan dressing compared to regular gauze-type surgical dressing in patients with surgical wounds. The chitosan dressing protected the wound from potential infection by microbes such as staphylococci, *Enterococcus* spp., *Enterobacter* spp., and *Parabacteroides*, as well as improved the composition of probiotic microbes, including *Prevotella*, *Lactobacillus*, and *Oscillibacter*, for stimulating skin immunity and wound healing. These findings suggest that chitosan dressing not only acts as an effective antimicrobial and procoagulant but also promotes wound repair by providing beneficial microbiota. Hence, chitosan dressing could be suitable as a first-line intervention for wound management.

## Data Availability

Data are available upon request.

## Abbreviations

ECM	Extracellular matrix
PT	Prothrombin time
aPTT	Activated partial thromboplastin time
ATP	Adenosine triphosphate
PLS-DA	Partial least squares discriminant analysis
FTIR	Fourier transform infra-red
SEM	Scanning electron microscopy
DMSO	Dimethyl sulfoxide
RFU	Relative fluorescence units
DNA	Deoxyribonucleic acid
rRNA	Ribosomal ribonucleic acid
PCR	Polymerase chain reaction
ANOVA	Analysis of variance
ANOSIM	Analysis of similarities
MRPP	Multiple Response Permutation Procedures
DMEM	Dulbecco’s modified Eagle’s medium
PBS	Phosphate-buffered saline

## References

[1] Oryan A., Alidadi S., Moshiri A., Maffulli N. (2014). Bone regenerative medicine: Classic options, novel strategies, and future directions. J. Orthop. Surg. Res..

[2] Sivashankari P.R., Prabaharan M. (2016). Prospects of chitosan-based scaffolds for growth factor release in tissue engineering. Int. J. Biol. Macromol..

[3] Sultankulov B., Berillo D., Sultankulova K., Tokay T., Saparov A. (2019). Progress in the Development of Chitosan-Based Biomaterials for Tissue Engineering and Regenerative Medicine. Biomolecules.

[4] Celikkin N., Rinoldi C., Costantini M., Trombetta M., Rainer A., Święszkowski W. (2017). Naturally derived proteins and glycosaminoglycan scaffolds for tissue engineering applications. Mater. Sci. Eng. C.

[5] Tharanathan R.N., Kittur F.S. (2003). Chitin—the undisputed biomolecule of great potential. Crit. Rev. Food Sci. Nutr..

[6] He Q., Gong K., Ao Q., Ma T., Yan Y., Gong Y., Zhang X. (2013). Positive charge of chitosan retards blood coagulation on chitosan films. J. Biomater. Appl..

[7] Jayakumar R., Prabaharan M., Kumar P.T.S., Nair S.V., Tamura H. (2011). Biomaterials based on chitin and chitosan in wound dressing applications. Biotechnol. Adv..

[8] Liu H., Wang C., Li C., Qin Y., Wang Z., Yang F., Li Z., Wang J. (2018). A functional chitosan-based hydrogel as a wound dressing and drug delivery system in the treatment of wound healing. RSC Adv..

[9] Grifoll-Romero L., Pascual S., Aragunde H., Biarnés X., Planas A. (2018). Chitin Deacetylases: Structures, Specificities, and Biotech Applications. Polymers.

[10] Clark R.A.F., Lanza R., Langer R., Vacanti J. (1997). Principles of Tissue Engineering: Wound Repair: Basic Biology to Tissue Engineering.

[11] Healy B., Freedman A. (2006). Infections. BMJ.

[12] Gould I.M., David M.Z., Esposito S., Garau J., Lina G., Mazzei T., Peters G. (2012). New insights into meticillin-resistant Staphylococcus aureus (MRSA) pathogenesis, treatment and resistance. Int. J. Antimicrob. Agents.

[13] Abdelrahman T., Newton H. (2011). Wound dressings: Principles and practice. Surgery.

[14] Potara M., Jakab E., Damert A., Popescu O., Canpean V., Astilean S. (2011). Synergistic antibacterial activity of chitosan-silver nanocomposites on Staphylococcus aureus. Nanotechnology.

[15] Ing L.Y., Zin N.M., Sarwar A., Katas H. (2012). Antifungal activity of chitosan nanoparticles and correlation with their physical properties. Int. J. Biomater..

[16] Landriscina A., Rosen J., Friedman A.J. (2015). Biodegradable chitosan nanoparticles in drug delivery for infectious disease. Nanomedicine.

[17] Matica M.A., Aachmann F.L., Tøndervik A., Sletta H., Ostafe V. (2019). Chitosan as a Wound Dressing Starting Material: Antimicrobial Properties and Mode of Action. Int. J. Mol. Sci..

[18] Dai T., Tanaka M., Huang Y.Y., Hamblin M.R. (2011). Chitosan preparations for wounds and burns: Antimicrobial and wound-healing effects. Expert Rev. Anti Infect. Ther..

[19] Wang Y.W., Liu C.C., Cherng J.H., Lin C.S., Chang S.J., Hong Z.J., Liu C.C., Chiu Y.K., Hsu S.D., Chang A.H. (2019). Biological effects of chitosan-based dressing on hemostasis mechanism. Polymers.

[20] Kong M., Chen X.G., Xing K., Park H.J. (2010). Antimicrobial properties of chitosan and mode of action: A state of the art review. Int. J. Food Microbiol..

[21] Wang Y.H., Liu C.C., Cherng J.H., Fan G.Y., Wang Y.W., Chang S.J., Hong Z.J., Lin Y.C., Hsu S.D. (2019). Evaluation of chitosan-based dressings in a swine model of artery-injury-related shock. Sci. Rep..

[22] Khan W.S., Pyarasani S., Asmatulu R. (2020). Reinforcing antibacterial hydrogels through electrospun nanofiber layers for soft tissue engineering. J. Polym. Res..

[23] Menezes J.E.S.A., dos Santosa H.S., Ferreira M.K.A., Magalhães F.E.A., da Silva D.S., Bandeira P.N., Saraiva G.D., Pessoa O.D.L., Ricardo N.M.P.S., Cruz B.G. (2020). Preparation, structural and spectroscopic characterization of chitosan membranes containing allantoin. J. Mol. Struct..

[24] Poonguzhali R., Basha S.K., Kumari V.S. (2017). Synthesis and characterization of chitosan-PVP-nanocellulose composites for in-vitro wound dressing application. Int. J. Biol. Macromol..

[25] Fernandes Queiroz M., Melo K.R., Sabry D.A., Sassaki G.L., Rocha H.A. (2014). Does the use of chitosan contribute to oxalate kidney stone formation?. Mar. Drugs.

[26] Zahedi P., Rezaeian I., Ranaei-Siadat S.-O., Jafari S.-H., Supaphol P. (2010). A review on wound dressings with an emphasis on electrospun nanofibrous polymeric bandages. Polym. Adv. Technol..

[27] Monroe D.M., Hoffman M. (2012). The clotting system—A major player in wound healing. Haemophilia.

[28] Raber M.N., Walker H.K., Hall W.D., Hurst J.W. (1990). Coagulation Tests. Clinical Methods: The History, Physical, and Laboratory Examinations.

[29] de Lima J.M., Sarmento R.R., de Souza J.R., Brayner F.A., Feitosa A.P., Padilha R., Alves L.C., Porto I.J., Batista R.F., de Oliveira J.E. (2015). Evaluation of hemagglutination activity of chitosan nanoparticles using human erythrocytes. Biomed. Res. Int..

[30] Chen L., Tianqing L. (2008). Interaction behaviors between chitosan and hemoglobin. Int. J. Biol. Macromol..

[31] Okamoto Y., Yano R., Miyatake K., Tomohiro I., Shigemasa Y., Minami S. (2003). Effects of chitin and chitosan on blood coagulation. Carbohydr. Polym..

[32] Percival S.L. (2017). Importance of biofilm formation in surgical infection. Br. J. Surg..

[33] Bowler P.G., Duerden B.I., Armstrong D.G. (2001). Wound microbiology and associated approaches to wound management. Clin. Microbiol. Rev..

[34] Li P., Poon Y.F., Li W., Zhu H.Y., Yeap S.H., Cao Y., Qi X., Zhou C., Lamrani M., Beuerman R.W. (2011). A polycationic antimicrobial and biocompatible hydrogel with microbe membrane suctioning ability. Nat. Mater..

[35] Raafat D., von Bargen K., Haas A., Sahl H.G. (2008). Insights into the mode of action of chitosan as an antibacterial compound. Appl. Environ. Microbiol..

[36] Rajkumari N., Mathur P., Misra M.C. (2014). Soft Tissue and Wound Infections Due to Enterococcus spp. Among Hospitalized Trauma Patients in a Developing Country. J. Glob. Infect. Dis..

[37] Mangram A.J., Horan T.C., Pearson M.L., Silver L.C., Jarvis W.R. (1999). Guideline for Prevention of Surgical Site Infection, 1999. Centers for Disease Control and Prevention (CDC) Hospital Infection Control Practices Advisory Committee. Am. J. Infect. Control..

[38] Nagy E., Urbán E., Nord C.E. (2011). ESCMID Study Group on Antimicrobial Resistance in Anaerobic Bacteria. Antimicrobial susceptibility of Bacteroides fragilis group isolates in Europe: 20 years of experience. Clin. Microbiol. Infect..

[39] Tsiouris C.G., Tsiouri M.G. (2017). Human microflora, probiotics and wound healing. Wound Med..

[40] Reid G., Jass J., Sebulsky M.T., McCormick J.K. (2003). Potential uses of probiotics in clinical practice. Clin. Microbiol. Rev..

[41] Singh D., Lee C.H. (2018). Intraspecies Volatile Interactions Affect Growth Rates and Exometabolomes in *Aspergillus oryzae* KCCM 60345. J. Microbiol. Biotechnol..

[42] Hou L., Wang J., Zhang W., Quan R., Wang D., Zhu S., Jiang H., Wei L., Liu J. (2020). Dynamic Alterations of Gut Microbiota in Porcine Circovirus Type 3-Infected Piglets. Front. Microbiol..

[43] Chijiiwa R., Hosokawa M., Kogawa M., Nishikawa Y., Ide K., Sakanashi C., Takahashi K., Takeyama H. (2020). Single-cell genomics of uncultured bacteria reveals dietary fiber responders in the mouse gut microbiota. Microbiome.

[44] Magoč T., Salzberg S.L. (2011). FLASH: Fast length adjustment of short reads to improve genome assemblies. Bioinformatics.

[45] Caporaso J.G., Kuczynski J., Stombaugh J., Bittinger K., Bushman F.D., Costello E.K., Fierer N., Peña A.G., Goodrich J.K., Gordon J.I. (2010). QIIME allows analysis of high-throughput community sequencing data. Nat. Methods.

[46] Paulson J.N., Stine O.C., Bravo H.C., Pop M. (2013). Differential abundance analysis for microbial marker-gene surveys. Nat. Methods.

